# Navigating Adolescence with PKU: Adherence, Metabolic Control, and Wellbeing in a UK Clinical Centre

**DOI:** 10.3390/nu17213409

**Published:** 2025-10-29

**Authors:** Alex Pinto, Anne Daly, Sharon Evans, Catherine Ashmore, Anita MacDonald

**Affiliations:** Birmingham Women’s and Children’s Hospital, Birmingham B4 6NH, UK; a.daly3@nhs.net (A.D.); evanss21@me.com (S.E.); catherine.ashmore@nhs.net (C.A.); anita.macdonald@nhs.net (A.M.)

**Keywords:** phenylketonuria, anxiety, depression, phenylalanine, adolescents

## Abstract

**Background**: During adolescence, the brain is undergoing anatomical and physiological maturation processes with changes to cognitive development. However, in adolescents with phenylketonuria (PKU), executive function and mental health are adversely affected by high blood phenylalanine (Phe) levels. **Objectives**: We aim to describe clinical outcomes in adolescents with PKU. **Methods**: Cross-sectional questionnaires were performed on quality of life (EuroQol “EQ-5D-5L” questionnaire), anxiety and depression (Hospital Anxiety and Depression Scale- HADS) and food neophobia in a single PKU centre. Retrospective data on metabolic control (previous 12 months) and medical history, and current data on anthropometry, dietary treatment, and comorbidities were collected. **Results**: In total, 33 adolescents with PKU participated with a mean age of 13.5 ± 1.3 y (16 boys, 17 girls). All were on a Phe restricted diet, with 3 also prescribed sapropterin. Questionnaires were self-completed by *n* = 25/33 (76%) adolescents. A mean of 36 ± 26 blood Phe spots were performed over 12 months. There was a mean of 83% of blood Phe < 600 µmol/L and 49% < 360 µmol/L. In total, 39% (*n* = 13/33) of adolescents were overweight/obese, 18% (*n* = 6/33) overweight and 21% (*n* = 7/33) obese. Medical history documented mental health disorders (anxiety/depression) in 7 cases, low mood, suicidal thoughts and self-harming in 5, and neuro diversity in 4 (autism and/or attention-deficit hyperactivity disorder (ADHD). In the HADS questionnaire, 12% (*n* = 3/25) of adolescents scored borderline abnormal for anxiety and 12% (*n* = 3/25) abnormal for depression. Mean anxiety scores for females were almost twice as high compared to males. No significant correlation was found between blood Phe and anxiety/depression scores. However, higher Phe levels showed a trend towards reduced enjoyment and emotional responsiveness, including lower scores on measures of pleasure, cheerfulness, and emotional reactivity. Although not statistically significant, these findings suggest a potential subtle association between poorer metabolic control and mood disturbances in adolescents with PKU. No food neophobia was identified in our study. **Conclusions**: Adolescents with PKU presented with high levels of depression and anxiety. Long term studies focusing on quality of life and neurocognition even when achieving the PKU European guidelines are necessary. Different therapeutic options are needed to improve the outcome of patients with PKU.

## 1. Introduction

Phenylketonuria (PKU) is a rare inherited metabolic disorder resulting from pathogenic variants in the phenylalanine hydroxylase (PAH) gene, leading to markedly reduced or absent activity of the PAH enzyme. This enzymatic deficiency impairs the hydroxylation of phenylalanine (Phe) to tyrosine (Tyr), causing accumulation of Phe and its metabolites in the blood, brain and peripheral tissues [1]. At elevated concentrations, Phe crosses the blood–brain barrier, exerting a neurotoxic effect [2]. Normally, 10–20% of Phe supports protein synthesis; the rest is hydroxylated to Tyr. Tyr supports the synthesis of dopamine, adrenaline, and noradrenaline, enables melanin production via thyroxine conversion, and contributes to energy metabolism through breakdown into acetoacetate and fumarate. In PKU, excess Phe is diverted to form phenylketone bodies (e.g., phenylpyruvate), which are excreted in the urine [2,3].

High blood Phe can lead to a range of neurological symptoms including intellectual disability, hyperactivity, autistic-like behaviours, seizures, movement abnormalities, and aggression [4]. The underlying pathophysiology is multifaceted and is thought to involve both disruption of white matter and deficiencies in neurotransmitter synthesis. Brain myelination, a critical process for efficient neural communication, begins in late gestation and continues through adulthood [5]. Hyperphenylalaninemia adversely impacts this process impairing executive functions and overall cognitive performance [6]. Lifelong treatment is essential, and a Phe restricted diet supplemented with a low-Phe protein substitute is started immediately following newborn screening in infancy [7].

Maintaining dietary adherence in individuals becomes increasingly difficult with age. While most young children meet target blood Phe levels under parental supervision, adherence often declines from early adolescence [5,8] accompanied by lower clinic attendance [9,10]. At the same time, in adolescence the brain undergoes significant structural and functional maturation, characterized by anatomical and physiological brain remodelling [11]. Giedd et al. [12] demonstrated that substantial reorganization of neural structures occurs, with parallel refinement in cognitive function [11]. Key neurodevelopmental processes include synaptic pruning, increased white matter volume, and dynamic changes in neurotransmitter systems [13]. While the volume of brain tissue remains stable at this time, axonal myelination, particularly in the frontal cortex, continues well into adolescence [14]. Barnea-Goraly et al., using diffusion tensor imaging, reported widespread increases in white matter anisotropy across brain regions implicated in attention, motor function, memory, and cognitive control in individuals aged 6 to 19 years [15]. During adolescence, anisotropy increases due to progressive myelination and enhanced structural organization in white matter tracts [15]. The development of executive functions, defined as the capacity to coordinate and regulate cognition and behaviour, also progresses markedly during adolescence [16]. Advances are seen in inhibitory control [16,17], processing speed [18], working memory, and decision-making capabilities [19].

In adolescence, there is rapid physical, psychological, and emotional development. Key milestones include the maturation of personal identity, autonomy, emotional regulation, self-esteem, impulse control, and social interaction skills [20]. It is often a psychologically complex and emotionally charged stage of development. The prefrontal cortex remains functionally immature, contributing to an increased tendency for risk-taking behaviours, including unprotected sexual activity, substance use, and illegal driving [21]. Individuals may experience mood volatility, sleep disturbances, and a heightened sense of being misunderstood. For individuals with PKU, these normative developmental challenges may be further intensified by the demands of managing a chronic condition. Some adolescents with PKU may struggle with feelings of frustration, anger, or shame stemming from their diagnosis and dietary restrictions [22]. Acceptance of the condition can be difficult, and the need for lifelong treatment may lead to resentment or denial. Di Ciommo et al. [23] reported that the fear of social stigmatization, especially related to dietary limitations and the use of protein substitutes, undermine self-esteem and social confidence. Moreover, there may be inadequate disease-related knowledge and low awareness of how high blood Phe levels may contribute to symptoms such as fatigue, irritability, or headaches.

Adolescents with PKU may have more self-reported mental health issues than ‘healthy’ populations, with a higher rate of anxiety [24], self-harm or even suicidal thoughts. There may be more behavioural difficulties, interpersonal conflicts and strained relationships. Some may experience decline in academic performance or a lack of motivation to engage in schoolwork. These difficulties may be compounded by earlier patterns of overprotection, excessive attention and overindulgence from parents that may limit opportunities for them to develop independent problem-solving and conflict-resolution skills [25]. Overall, their social networks and connections may be insecure leading to low self-confidence, reduced emotional resilience, and a heightened risk of social isolation.

Disordered eating may be more evident during adolescence with disturbances in eating behaviours, appetite or food intake, complicating the individual’s relationship with food. Some may engage in restrictive eating due to concerns about body image, with girls commonly under more pressure than boys to have a lower body weight [26]. Others may overeat in response to emotional distress, although studies in adolescents with PKU show that the prevalence of overweight and obesity is similar to healthy adolescents and that it is not associated with the disease or dietary treatment [27]. Nevertheless, eating disorders may be underdiagnosed and unrecognized in adolescents with PKU due to inadequate screening practices and the absence of PKU specific, validated assessment tools for disordered eating [28].

Despite the existence of comprehensive European guidelines for PKU management [29], few studies have specifically examined clinical and psychosocial outcomes in adolescents receiving care aligned with these recommendations [30,31]. The guidelines, developed to standardise treatment and optimise neuropsychological outcomes across Europe, identify adolescence as a critical period, yet real-world evaluations of their impact in this age group remain limited. This observational, cross-sectional study aimed to evaluate clinical outcomes and metabolic control in adolescents with PKU receiving care at a single treatment centre.

## 2. Materials and Methods

### 2.1. Patient Selection

Inclusion criteria included adolescent patients with PKU, aged 12 to 17 years at the time of study commencement, identified via newborn screening and continuously treated from early infancy. All participants were under the ongoing care of a single PKU centre in the UK: Birmingham Children’s Hospital. 

Exclusion criteria included individuals with late-treated PKU, children < 12 years or adults aged ≥ 18 years at the start of the study period.

### 2.2. Study Design

**Figure 1:**
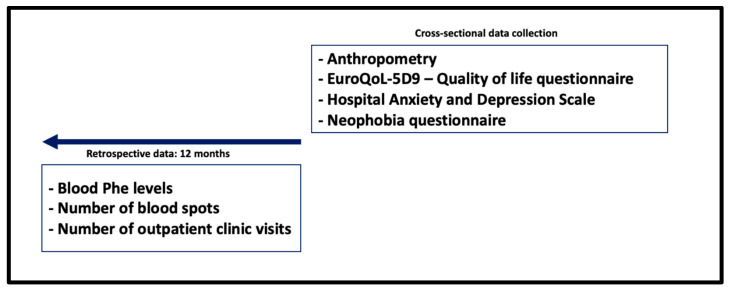
Study design.

The aim of this study was to examine blood Phe control, nutritional, physical and mental health outcomes of adolescents with PKU. Management practices were compared with the European PKU guidelines [29] as a standard of care. The study design is presented in Figure 1.

### 2.3. Usual Clinical Monitoring

Weekly blood spot Phe monitoring was encouraged, using filter paper cards (Perkin Elmer 226; UK Standard NBS, Public Health England, London, UK) collected at home by the patient or caregiver. Samples were posted to the hospital and analysed via tandem mass spectrometry (MS/MS). Blood Phe levels were evaluated against the recommended therapeutic range of 120–600 μmol/L, as outlined in the 2025 European Guidelines for the management of PKU [29]. Routine clinical follow-up included biannual outpatient visits.

### 2.4. Data Collection

Retrospective data were collected over 12 months from January 2020 to March 2021 on: blood Phe levels (last blood Phe level when questionnaires were completed), number of blood spots conducted, and the number of outpatients clinic visits attended. Cross-sectional data was collected on documented co-morbidities and anthropometry (weight, height and body mass index (BMI)) reported in the participants medical notes. Dietary treatment was recorded (number of protein/Phe exchanges [50 mg Phe = 1 g protein] prescribed), protein equivalent from protein substitute and type of protein substitute used. Overweight and obesity were classified according to WHO BMI-for-age standards, with overweight defined as a BMI z-score > +1 SD and obesity as >+2 SD relative to age- and sex-specific reference curves [30].

Patients self-completed questionnaires on quality of life (EuroQoL-5D9), anxiety and depression symptoms (Hospital Anxiety and Depression Scale—HADS) and food neophobia (attitudes towards food). The EuroQoL-5D-5L (EQ-5D-5L) a standardized instrument for assessing health-related quality of life (HRQoL), was comprised of five dimensions: mobility, self-care, usual activities, pain/discomfort, and anxiety/depression. Each dimension was rated on a five-level scale: no problems, slight problems, moderate problems, severe problems, and extreme problems, enabling nuanced classification of health states.

The Hospital Anxiety and Depression Scale (HADS), a validated screening tool, had two subscales: HADS-A (anxiety) and HADS-D (depression), and each was scored from 0 to 21. Classification thresholds were as follows: 0–7 normal, 8–10 borderline abnormal, 11–21 abnormal.

The Food Neophobia Scale (FNS) was a 10-item self-report instrument that evaluated attitudes toward unfamiliar foods, including reluctance to try new items and broader neophobic tendencies. Higher scores indicated greater neophobia, reflecting reduced willingness to engage with novel foods or experiences, while lower scores suggested increased openness to dietary and situational novelty.

### 2.5. Ethics

Ethical approval was obtained from the South Yorkshire ethical committee on the 29th of September 2021 (REC number: 21/YH/0070). Patients and parents/caregivers signed informed consent/assent depending on age, prior to starting the study. Good Clinical Practice guidelines and the principles of the “Declaration of Helsinki” (52nd WMA General Assembly, Edinburgh, Scotland, 3–7 October 2000) were used during this study.

### 2.6. Statistical Analysis

No formal sample size calculation was performed, as all patients meeting the inclusion criteria were recruited for this study. Statistical significance was defined as *p* < 0.05. Quantitative data and continuous variables were summarized using mean ± standard deviation (SD), while categorical variables were presented as absolute frequencies and percentages. Differences in food neophobia questionnaire scores between male and female participants were obtained by fitting generalized linear model with Poisson link and a fixed effect for gender. All statistical analyses were conducted using GraphPad Prism (Version 10.1.0, 18 October 2023; GraphPad Software, Boston, MA, USA).

## 3. Results

### 3.1. Participants

A total of 33 patients with PKU were included in the study: 17 females and 16 males. The overall mean age was 13.5 ± 1.3 years (range: 12–16 years). Female participants had a mean age of 13.0 ± 1.0 years (range: 12–15), and males had a mean age of 14.1 ± 1.4 years (range: 12–16).

Severity of individual PKU was classified according to their PAH gene variants. Where genotypic data were unavailable, classification was based on blood Phe levels at diagnosis. Nineteen patients were classified as classical PKU, 13 as mild PKU and 1 was without classification (Supplementary Table S1).

### 3.2. Dietary Management

All patients were prescribed a Phe-restricted diet that included avoidance of high-protein foods, use of an exchange system (1 g protein ≈ 50 mg Phe) to allocate Phe according to individual tolerance, and supplementation with a low-Phe protein substitute. Low-protein foods, defined as those containing protein up to 0.5 g/100 g, or fruits and vegetables with a Phe content up to 75 mg/100 g, were permitted without measurement or restriction.

The mean number of 1 g protein exchanges (50 mg Phe) prescribed was 10.2 ± 8.6/day (11.9 ± 10.1 in males and 8.6 ± 6.9 in females). The mean prescription of protein equivalent from protein substitute was 64.8 ± 12.3 g/day (males: 66.8 ± 10.1; females: 62.9 ± 14.0). Mean total protein intake was 75.4 ± 11.0 g/day (males: 79.4 ± 10.1; females: 71.6 ± 10.8) or 1.4 ± 0.3 g/kg/day (males: 1.5 ± 0.3; females: 1.4 ± 0.3).

Nine participants were prescribed glycomacropeptide (GMP)-based protein substitutes, and 24 received amino acid (AA)-based substitutes in ready-to-use pouches. All patients had access to special low-protein foods provided through the National Health Service (NHS). Protein substitutes and special low-protein foods were prescribed by the community general practitioner (GP) and supplied via a home delivery service.

Three participants were prescribed sapropterin (one male and two females) at 20 mg/kg, taken once daily with food. At the time of study this was only available through research studies.

### 3.3. History from Medical Notes

Five participants (15%, n = 5/33) had a school educational health care plan and n = 2/33 (6%) attended a special school due to autism as a co-morbidity. Twelve percent (n = 4/33) had autism and/or attention deficit hyperactivity disorder (ADHD), 21% (n = 7/33) depression, 21% anxiety (n = 7/33) and 15% (n = 5/33) had low mood with suicidal or self-harm thoughts. Recurrent constipation (n = 1), school anxiety (n = 1) and behaviour issues (n = 1) were documented in the medical notes. Methylphenidate for ADHD treatment (n = 1), laxatives (n = 1) and anti-depressants (n = 2) were prescribed medications.

### 3.4. Anthropometry

The mean weight and height of participants were 55.2 ± 7.8 kg and 158.3 ± 8.3 cm, respectively. The mean body mass index (BMI) was 21.2 ± 2.7, with a corresponding mean BMI z-score of 0.95 ± 0.94. Overall, 39% (n = 13/33) of participants were classified as overweight or obese, with 18% (n = 6/33) overweight and 21% (n = 7/33) obese. Among male participants, 25% (n = 4/25) were overweight and 13% (n = 2/16) were obese. In females, 53% (n = 9/17) were overweight or obese (24% (n = 4/17) overweight and 29% (n = 5/17) obese). Table 1 presents blood Phe control and the anthropometry of adolescents participating in this study. Anthropometry was assessed at clinic visits.

### 3.5. Blood Phenylalanine Control

Over a 12-month period, participants submitted a mean of 36 ± 26 dried blood spot samples for Phe analysis, representing 69% of the expected 52 samples. A mean of 83% of reported Phe levels were <600 µmol/L, and 49% were <360 µmol/L. Table 1 presents blood Phe control and anthropometrics of adolescents participating in this study.

### 3.6. Neophobia Questionnaire

A food neophobia questionnaire was completed by 76% of participants (n = 25/33) (Table 2). The instrument assessed both food-specific and general neophobic tendencies, with responses rated on a 7-point Likert scale ranging from 1 (always) to 7 (never). Supplementary Table S2 summarizes the results for the 25 respondents. Overall, the cohort did not exhibit strong indicators of food neophobia. However, female participants demonstrated significantly higher neophobic behaviour compared to males, reflected in lower mean scores (3.5 vs. 4.1 *p =* 0.012).

### 3.7. Hospital Anxiety and Depression Scale-HADS Questionnaire

The HADS tool was used to assess symptoms of anxiety and depression in participants. Of the 25 adolescents who completed the questionnaire, 12% (n = 3) scored in the abnormal range for depression, and an additional 12% (n = 3) scored in the boderline abnormal range. For anxiety, 8% (n = 2) were classified as borderline abnormal, while 40% (n = 10) scored in the abnormal range. Notably, mean anxiety scores were nearly twice as high in female participants compared to males (Table 3).

**Figure 2:**
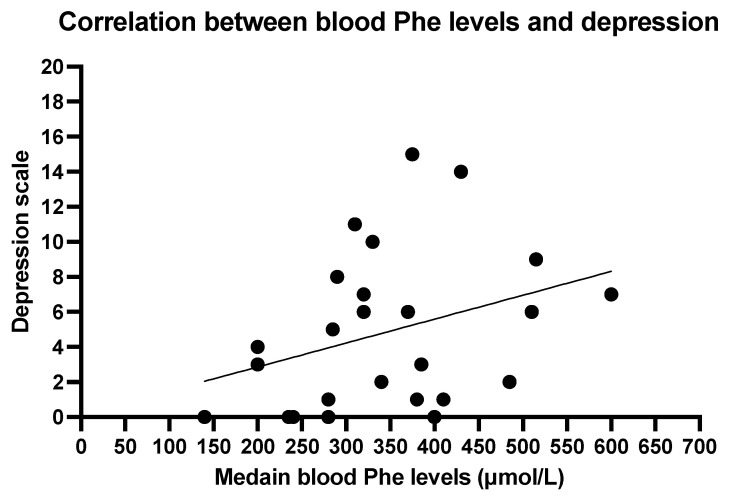
Correlation between blood phenylalanine levels and depression. Spearman R 0.3713, *p* 0.0677.

**Figure 3:**
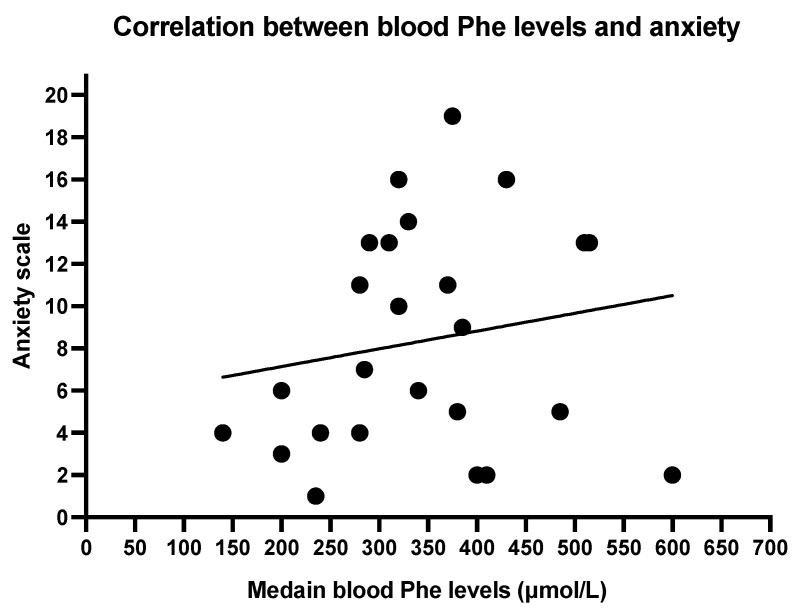
Correlation between blood phenylalanine levels and anxiety. Spearman R 0.1942, *p* 0.3523.

The Spearmen correlation between mean blood Phe levels and depression scale shows a weak correlation (r = 0.3713, *p* = 0.0677; Figure 2). No significant correlation was found between anxiety and mean blood Phe (r = 0.1942, *p* = 0.3523; Figure 3).

Table 4 presents a comparison between the mean of the three most recent blood Phe levels and individual item scores from the HADS tool. Although overall *p*-values were >0.05, indicating no statistically significant associations, a consistent trend was observed: higher blood Phe levels appeared to correlate with elevated scores on several HADS items. This trend was particularly evident in responses to the following questions: “I still enjoy the things I used to enjoy;” “I can laugh and see the funny side of things;” “I feel cheerful;” “I get a sort of frightened feeling like butterflies in the stomach;” “I can enjoy a good book, radio or TV program.”

### 3.8. Euroqol-5D-5L (EQ-5D-5L)–Visual Analogue Scale

The EQ-5D-5L scale described 5 dimensions (mobility, selfcare, usual activities, pain/discomfort and anxiety/depression) with a maximum general health status classification score of 100 perceived by patients. Overall, 76% (n = 25/33) of the participants completed the questionnaire, with a mean total score of 82.1 ± 15.3 (86.8 ± 15.3 in males and 82.1 ± 15.3 in females).

For usual activities (work, study, leisure), 20% (n = 5/25) reported slight problems and one participant reported moderate problems. Twenty-four per cent (n = 6/25) reported slight pain and discomfort. This scale also evaluated feelings of anxiety and depression with 20% (n = 5/25) describing moderate anxiety and depression, 16% (n = 4/25) severe, and 1 patient had extreme feelings of anxiety and depression.

### 3.9. Hospital Resources and Practices

Adolescents and their parents were reviewed by a specialist IMD multidisciplinary team consisting of a physician, specialist dietitian and nurse. They had access to a clinical psychologist, but only by referral in response to a specific clinical need, with 30% (n = 10/33) of patients seen at least once by a psychologist and 24% (n = 8/33) by a counsellor/support worker in the previous 12 months. Participants began a transition process at the age of 12 years, with preparation leading to transfer to adult clinic care between the ages of 16 and 17 years.

## 4. Discussion

This study evaluated outcomes among adolescents with PKU attending follow-up care at one specialist centre. Metabolic control was satisfactory, with a mean of three blood Phe tests per month, 83% of Phe levels < 600 µmol/L, and 49% < 360 µmol/L. However, a high prevalence of anxiety and depression was observed, particularly among females, who reported anxiety symptoms at twice the rate of males. Mental health outcomes did not correlate strongly with blood Phe concentrations, yet questionnaire data aligned with documented psychological histories, highlighting the need for enhanced psychosocial support that was currently below the standards recommended by the European PKU clinical guidelines [29]. Elevated rates of overweight and obesity were also noted, raising concerns regarding dietary balance and energy intake. No significant indicators of food neophobia were identified, suggesting a general openness to novel dietary options despite longstanding dietary restrictions.

The majority of patients followed a Phe restriction combined with a low-Phe protein substitute, with only 3 adolescents prescribed adjunct pharmaceutical treatment (sapropterin). The dietary regimen placed a significant burden on adolescents, and for many it was incompatible with their lifestyle. Although pharmaceutical treatments offer increased dietary Phe tolerance and lower blood Phe levels, oral drugs remain underutilized with inconsistent access across healthcare systems. A multicentre European study [10] involving 1323 patients reported that only 17% were treated with sapropterin, although at least 30% of the PKU population could potentially gain benefit. Similarly, nationwide data from France indicated that only 5% of 1911 adult patients were prescribed sapropterin [31]. These figures highlight a substantial gap between clinical potential and real-world application of pharmacological treatments in PKU.

Despite our centre’s longstanding expertise in PKU management and the presence of a well-established multidisciplinary team, routine neurocognitive assessments, as recommended by the European PKU guidelines [29], have not been systematically integrated into standard care pathways. Psychological support has similarly remained reactive, typically initiated through specific clinical referrals rather than embedded within a proactive, preventative framework. Over the past year, approximately 30% of the cohort accessed psychological services; however, these interactions were predominantly prompted by acute mental health concerns rather than routine screening or early intervention.

Psychologists are integral to the comprehensive care of individuals with PKU and should be involved in patient care from the point of diagnosis [30]. In adolescent care, their role is particularly important: psychologists help patients identify personal challenges, foster insight, develop coping strategies, and build self-advocacy skills. Through establishing a safe and trusting therapeutic relationship, they support adolescents in navigating the complex demands of dietary management, academic pressures, and evolving personal goals [32]. Current practice across UK centres falls short of the standards outlined in the European PKU guidelines [29], particularly in the routine integration of psychological care. This represents a clear gap in service provision and highlights the need for systemic change. Longitudinal studies evaluating the impact of sustained psychological support on quality of life and metabolic control are urgently needed to inform best practice. To address this disparity, professional groups and patient advocacy organisations in the UK must work collaboratively to urge the NHS to adopt and implement guideline-concordant care.

Emotional disorders are common during adolescence, with anxiety and depression particularly prevalent [33]. These conditions are frequently associated with impaired school attendance, academic performance, concentration, and daily functioning. Emotional distress may lead to social withdrawal, exacerbating feelings of isolation and loneliness [34]. In more severe cases, depressive symptoms can result in self-harm or suicidal ideation, emphasizing the critical importance of early identification and intervention. Behavioural disorders may further compound risk, potentially contributing to antisocial behaviour or criminal activity [35]. In our cohort, 21% of adolescents had a documented history of depression and anxiety, and 15% had self-harmed or experienced suicidal thoughts. These figures exceed those reported in the general adolescent population. Among adolescents without chronic health conditions, approximately one in seven aged 10–19 years experience a mental health disorder, with anxiety affecting 4.4% of 10–14-year-olds and 5.5% of 15–19-year-olds. Data from the 2017 NHS survey in England indicated an age-related increase in depressive disorder prevalence, rising from 0.3% in children aged 5–10 years to 2.7% in those aged 11–16 years, notably lower than rates observed in our PKU cohort [36]. Sex differences in depression prevalence also warrant consideration. Hetrick et al. [37] reported no significant differences prior to puberty, but from age 12 onwards, rates in females were approximately twice those in males, consistent with our observations. Failure to address mental health conditions during adolescence in individuals with PKU may have long-term consequences, impairing both physical and psychological health and limiting opportunities for personal fulfillment in adulthood [38]. Although previous studies, such as Charrière et al. [31], have shown higher rates of depression in individuals with PKU over the age of 16 compared to the general population, data remain limited. Our findings align with these trends; however, the cross-sectional design of our study precludes conclusions about causality. The elevated prevalence of depression and anxiety may be attributable to the burden of living with a chronic condition, but could also reflect broader developmental vulnerabilities during adolescence, including social pressures, emotional volatility, and hormonal changes.

Overall, we did not find any relation between blood Phe levels and anxiety and depression, although trends were found towards an association between higher blood Phe and lower scores on statements such as “I still enjoy the things I used to enjoy”, “I can laugh and see the funny side of things”, “I feel cheerful”, “I get a sort of frightened feeling like butterflies in the stomach” and “I can enjoy a good book, radio or TV program.” These items reflect aspects of anhedonia, emotional reactivity, and general affective wellbeing. While not reaching statistical significance, the observed pattern may suggest a subtle relationship between metabolic control and mood-related experiences. To better understand these associations, future multicentre studies are needed to assess the prevalence of anxiety and depression across larger PKU populations, stratified by age and compared with matched controls from the general population. Such research is essential to inform targeted interventions and ensure that mental health support is appropriately integrated into routine PKU care.

As adolescence is a critical period of continued brain development and maturation, maintaining good blood Phe control should remain a priority [29]. In our cohort, adolescents were advised to perform weekly blood Phe monitoring, exceeding the monthly frequency recommended by European PKU guidelines. This increased monitoring was associated with acceptable metabolic control, with 83% of patients maintaining Phe levels below 600 µmol/L, consistent with findings by Pinto et al. [10]. We report mean blood Phe levels over a 12-month period, as longitudinal sampling offers a more representative measure of overall metabolic control than reliance on a single time-point, although blood Phe levels were included from the study’s data collection window. Notably, evidence suggests that variability in Phe levels over time may exert a greater influence on neurocognitive outcomes than concurrent Phe levels, particularly with increasing age [39].

Studies by Jahja and Manti suggest that neurocognitive outcomes are significantly better when Phe levels were consistently <240 µmol/L [24,40]. This raises important questions about if the upper target of 600 µmol/L remains appropriate, and if a universal threshold < 360 µmol/L should be considered for all age groups. Data on adolescent neurocognition in PKU remain limited, but recent systematic reviews by Romani et al. [6], Thomas et al. [32], and De Giorgi [8] show consistent impairments in reasoning, attention, visual–motor integration, IQ, and executive function. Crucially, better outcomes are associated with lower blood Phe control. The USA PKU Guidelines already recommend a universal upper limit < 360 µmol/L for all age groups [33]. Neurocognitive testing was not incorporated into this study, limiting the ability to draw definitive conclusions regarding the direct impact of metabolic control on objective cognitive performance. The self-reported questionnaires employed primarily captured subjective experiences rather than validated neuropsychological function. Consequently, the findings may be subject to bias, as individual responses are influenced by personal perception, which may itself be affected by elevated blood Phe levels. Given the established effects of suboptimal Phe control on cognitive processing, it is plausible that participants’ interpretations of questionnaire items were shaped by underlying neurocognitive changes not directly assessed in this study. This introduces a potential confound, whereby affective symptoms may reflect neurocognitive impairment rather than emotional distress alone, raising the risk of misattributing neuropsychological manifestations to psychological states without accounting for their possible metabolic origin.

The adolescents in our cohort were asked to describe their physical health. Twenty per cent reported minor difficulties with daily activities such as work, study, or leisure, while 24% experienced mild pain or discomfort. These findings are consistent with previous research by Bilder et al. [34], who documented increased rates of fatigue, sleep disturbances, and migraines among individuals with PKU when compared to the general population. Similarly, Quinn noted a range of physical symptoms in adults with PKU, including headaches, sleep difficulties, dermatological issues, gastrointestinal complaints, joint pain, and persistent hunger [35]. Kenneson also reported a high prevalence of skin disorders among both children and adults with PKU [36].

In our cohort, the prevalence of overweight and obesity was high at 39%. This is concerning, as adolescent obesity is a strong predictor of adult obesity, a major, independent risk factor for cardiovascular disease [38,39]. Research has shown that cardiovascular risk in young adulthood correlates closely with levels of adiposity established in childhood [41]. The 2022 Health Survey for England reported a combined overweight and obesity prevalence of only 28% among children aged 11–15, with 9% classified as overweight and 19% as obese [42], and this was lower than our cohort. Most individuals in our group had classical PKU and followed a very restrictive diet, with a high percentage of energy intake supplied by specialist low protein foods [7]. We did not collect data on physical activity and exercise participation. While Rodrigues et al. [43] found no significant difference in overall overweight and obesity rates between individuals with PKU and the general population, their subgroup analysis also indicated that patients with classical PKU had a higher BMI than healthy controls.

Food neophobia has been proposed as an evolutionary safeguard against the ingestion of potentially harmful substances [44]. In our cohort, trends in food and general neophobia were not pronounced. Most participants reported being “sometimes,” “unsure,” or “rarely” neophobic, with a tendency toward greater neophobic behavior. These findings align with previous research suggesting that food neophobia in PKU may be shaped more by dietary constraints than by parental influence or innate food preferences [45]. With the introduction of adjunct therapies such as pegvaliase, dietary intake has become increasingly liberalized, marked by a significant rise in protein intake, in some cases, complete discontinuation of the low-Phe restricted diet [46,47]. This shift has been associated with a gradual reduction in food neophobia over time [48,49]. However, patients treated with diet only may benefit from routine psychological support. Adolescence represents a particularly vulnerable period, during which body image concerns and the restrictive nature of the PKU diet may contribute to heightened food-related anxiety. Addressing these psychosocial factors may enhance social confidence and, ultimately, improve dietary adherence [49].

However, we did identify a statistically significant gender difference in food neophobia scores within our cohort (mean score: 3.5 in males vs. 4.1 in females; *p* = 0.012). Female participants demonstrated greater neophobic tendencies, particularly in response to items such as “I am afraid to eat things I have never had before,” “I avoid speaking to people I don’t know,” and “I don’t like sitting next to someone I don’t know.” These findings suggest heightened avoidance behaviours in unfamiliar dietary and social contexts among female patients. This contrasts with previous studies. Bugi et al. [50] reported no gender differences in food neophobia among individuals with PKU, while Tonon et al. [51] found neophobia to be more prevalent in male patients. Studies in the general population have similarly yielded inconsistent findings regarding gender-based differences [52,53]. It has been proposed that food neophobia may vary with age, with a higher prevalence in males during childhood and adulthood, and a decline in females as they age [53,54]. Psychosocial factors may contribute to the gender-specific trends observed in our cohort. Body image and appearance-related concerns, which are often more pronounced in adolescent females, may influence food-related anxiety and social avoidance [55]. Additionally, healthcare systems may place greater emphasis on reproductive health in female patients with PKU, potentially amplifying perceived pressure around dietary adherence and metabolic control during key developmental phases [56].

In order to improve the outcome of adolescents with PKU it is important that self-care is encouraged. Education should begin in childhood and evolve with the patient’s developmental stage, helping them understand the impact of high Phe levels on physical health, cognitive function, emotional regulation, psychosocial development, and mental wellbeing. Empowering patients from an early age should support treatment adherence, foster self-awareness, and prepare them for greater responsibility in managing their condition as they transition into adolescence and adulthood.

This study has several limitations. First, blood Phe levels were collected up to 12 months prior to questionnaire completion. Although concurrent Phe data were available at the time of questionnaire administration, this may have introduced bias, as fluctuations in Phe levels can significantly influence both symptom presentation and neurocognitive functioning. Additionally, symptom reporting may have been underestimated. Existing research suggests that individuals with elevated Phe levels may experience reduced self-awareness, limiting their ability to accurately identify or articulate psychological difficulties. Only 25 of the 33 eligible participants completed the questionnaires, which may reduce the representativeness of the findings. Furthermore, the questionnaires used may not have comprehensively captured overall wellbeing, and no formal neuropsychological assessments were conducted. Rates of anxiety and depression observed in the cohort may be influenced by a range of factors associated with adolescence, and causality related to PKU cannot be assumed. Dietary data reflected prescribed rather than actual intake of natural protein and protein substitutes, which may not accurately represent true dietary adherence or nutritional status. Previous work by our team demonstrated that actual natural protein intake can exceed prescribed amounts by approximately 167%, largely due to the inclusion of ‘free’ or non-measured foods permitted within the UK dietary system [57]. This discrepancy appears consistent across patients and, notably, has not been associated with adverse effects on metabolic control.

It is also important to acknowledge that this study was conducted within a single centre, which may limit the generalizability of findings to the broader adolescent PKU population. Socioeconomic background, family support, or adherence to nutritional treatment were not included and may have influenced the outcomes. Additionally, the absence of a control group of adolescents without PKU restricts comparative analysis. The cross-sectional design further limits the ability to establish causal relationships between blood Phe control and psychological outcomes such as anxiety, depression, or quality of life. To address these limitations, multicentre studies and controlled trials are needed to determine if similar dietary patterns, psychosocial challenges, and clinical outcomes are observed across diverse care settings. Future re-search should incorporate concurrent dietary intake data and blood Phe levels during neuropsychological assessments to enable a more comprehensive analysis of contributing variables.

## 5. Conclusions

Adolescents with PKU presented high levels of depression and anxiety compared with previous data on PKU and the general population. Longitudinal studies are needed to evaluate quality of life and neurocognitive outcomes over time. Enhanced psychological support should be integrated into routine care to assist patients and families in managing the emotional and practical demands of lifelong dietary treatment. Furthermore, expanding access to alternative therapeutic options, including pharmacological and dietary innovations, will be critical to improving long-term outcomes in adolescents with PKU.

## Figures and Tables

**Table 1:** Blood phenylalanine control and anthropometrics of patients during 12 months.

	Patient’s Age (y)	Height (cm)	Weight (kg)	BMI (kg/m^2^)	Blood Phe Levels (μmol/L) over 1 Year	% of Blood Phe Levels < 600 μmol/L over 1 Year	% of Blood Phe Levels < 360 μmol/L over 1 Year	Number of Blood Spots for Phe Levels Returned over 1 Year
Total (mean ± SD)	13.5 ± 1.3	158.3 ± 8.3	55.2 ± 7.8	21.2 ± 2.7	405 ± 105	83 ± 30	49 ± 33	36 ± 26
Male (mean ± SD)	14.1 ± 1.4	162.9 ± 9.2	158.3 ± 8.3	20.6 ± 2.3	473 ± 226	73 ± 39	41 ± 35	28 ± 20
Female (mean ± SD)	13.0 ± 1.0	154.9 ± 5.0	53.8 ± 8.8	22.4 ± 3.4	341 ± 107	94 ± 8	56 ± 29	44 ± 29

Abbreviations: SD, standard deviation; y, years old; Phe, Phenylalanine, BMI, Body mass index.

**Table 2:** Food and general neophobia questionnaire results from 25 patients.

Food Neophobia	Total (n = 25)	Male (n = 10)	Female (n = 15)	*p*-Value
I frequently try new and different foods	3.9 ± 1.8	3.7 ± 1.8	4.1 ± 1.8	0.65
I don’t trust new foods	4.1 ± 1.9	4.4 ± 1.6	3.9 ± 2.1	0.518
I like foods from different countries	3.4 ± 1.9	3.9 ± 1.8	3.1 ± 2.0	0.312
I think ethnic food looks too weird to eat	4.5 ± 2.1	4.6 ± 2.2	4.4 ± 2.2	0.817
At social events, I will try new foods	3.4 ± 2.1	2.9 ± 2.0	3.7 ± 2.2	0.307
I am afraid to eat things I have never had before	4.0 ± 2.1	5.3 ± 1.8	3.1 ± 1.8	0.009
I am very particular about the foods I will eat	3.2 ± 2.1	3.3 ± 1.9	3.2 ± 2.3	0.892
I will eat almost anything	4.2 ± 2.3	4.4 ± 2.0	4.1 ± 2.6	0.69
I like to try new foods when eating out	3.6 ± 2.1	3.2 ± 2.2	3.9 ± 2.0	0.39
**General Neophobia**				
I am uncomfortable in new & different situations	3.4 ± 1.8	4.2 ± 1.6	2.9 ± 1.8	0.096
I prefer to be at home among familiar surroundings	3.6 ± 2.1	3.9 ± 1.9	3.3 ± 2.3	0.462
I avoid speaking to people I don’t know	3.1 ± 2.1	4.0 ± 2.2	2.5 ± 2.0	0.044
I feel uneasy in unfamiliar surroundings	3.6 ± 2.1	4.3 ± 1.7	3.1 ± 2.3	0.134
I don’t like sitting next to someone I don’t know	3.7 ± 2.5	4.7 ± 2.2	3.1 ± 2.5	0.04

Scale: 1 = always; 2 = mostly; 3 = sometimes; 4 = unsure; 5 = rarely; 6 = almost never; 7 = never. *p*-values obtained from fitting generalized linear model with Poisson link and a fixed effect for gender.

**Table 3:** HADS results presented for 25 patients participating in the study.

HADS Scale	Total	Male	Female
Anxiety (mean ± SD)	8.4 ± 5.3	5.4 ± 3.7	10.3 ± 5.3
Depression (mean ± SD)	4.8 ± 5.3	3.7 ± 3.6	5.6 ± 4.8

Normal Score on HADS 0–7; Borderline Abnormal Score 8-10; Abnormal Score 11–21 Abbreviations: HADS, Hospital Anxiety and Depression Scale; SD, standard deviation.

**Table 4:** Blood phenylalanine levels compared with HADS scores.

HADS Questions	Blood Phe Levels (Average Last 3 Levels) Per HADS Score (μmol/L)				*p*-Value
	HADS Score 0	HADS Score 1	HADS Score 2	HADS Score 3	
I feel tense or wound up 0-Not at all; 1-From time to time, occasionally; 2-A lot of the time; 3-Most of the time.	293 (235, 530)	405 (277, 486)	335 (320, 361)	352 (223, 501)	0.983
I still enjoy the things I used to enjoy 0-Definitely as much; 1-Not quite so much; 2-Only a little; 3-Hardly at all.	378 (232, 486)	327 (268, 361)	360 (360, 360)	522 (501, 543)	0.439
I get a sort of frightened feeling as if something bad is about to happen 0-Not at all; 1-A little, but it doesn’t worry me; 2-Yes, but not too badly; 3-Very definitely and quite badly.	372 (258, 472)	306 (220, 333)	457 (361, 535)	428 (394, 463)	0.285
I can laugh and see the funny side of things 0-As much as I always could; 1-Not quite so much now; 2-Definitely not so much now; 3-Not at all.	392 (258, 489)	272 (223, 324)	357 (318, 395)	480 (420, 522)	0.313
Worrying thoughts go through my mind 0-Only occasionally; 1-From time to time, but not too often; 2-A lot of the time; 3-A great deal of the time.	307 (199, 460)	333 (270, 485)	385 (323, 445)	428 (326, 513)	0.722
I feel cheerful 0-Most of the times; 1-Sometimes; 2-Not often; 3-Not at all.	385 (242, 485)	319 (266, 343)	465 (449, 481)	563 (563, 563)	0.251
I can sit at ease and feel relaxed 0-Definitely; 1-Usually; 2-Not often; 3-Not at all.	480 (372, 492)	320 (227, 422)	306 (280, 333)	498 (466, 531)	0.281
I feel as if I am slowed down 0-Not all; 1-Sometimes; 2-Very often; 3-Nearly all the time.	313 (273, 460)	415 (230, 556)	428 (394, 463)	337 (280, 433)	0.615
I get a sort of frightened feeling like butterflies in the stomach 0-Not all; 1-Ocassionally; 2-Quite often; 3-Very often.	372 (273, 478)	320 (223, 483)	360 (337, 433)	563 (563, 563)	0.46
I have lost interest in my appearance 0-I take just as much care as ever; 1-I may not take quite as much care; 2-I don’t take as much care as I should; 3-Definitely.	360 (293, 483)	385 (231, 472)	448 (391, 506)	173 (120, 227)	0.301
I feel restless as I have to be on the move 0-Not all; 1-Not very much; 2-Quite a lot; 3-Very much indeed.	313 (149, 458)	315 (258, 489)	360 (313, 488)	327 (273, 380)	0.585
I look forward with enjoyment to things 0-As much as I ever didl; 1-Rather less than I used to; 2-Definitely less than I used to; 3-Hardly at all.	287 (226, 473)	400 (334, 481)	340 (330, 350)	563 (498, 665)	0.132
I get sudden feelings of panic 0-Not all; 1-Not very often; 2-Quite often; 3-Very often indeed.	473 (336, 493)	293 (223, 421)	433 (360, 480)	308 (266, 393)	0.427
I can enjoy a good book, radio or TV program 0-Often; 1-Sometimes; 2-Not often; 3-Very seldom.	337 (250, 482)	300 (265, 362)	433 (433, 433)	767 (767, 767)	0.372

Abbreviations: Phe, Phenylalalnine; HADS, Hospital Anxiety and Depression Scale.

## Data Availability

The original contributions presented in this study are included in the article. Further inquiries can be directed to the corresponding author.

## Supplementary Materials

The following supporting information can be downloaded at: https://www.mdpi.com/article/10.3390/nu17213409/s1, Table S1: PKU severity of patients participating in study; Table S2: Food neophobia questionnaire results of all the participants in the in study.
