# Supplementary material for: Navigating Adolescence with PKU: Adherence, Metabolic Control, and Wellbeing in a UK Clinical Centre
Source: Nutrients. 2025 Oct 29;17(21):3409. doi: 10.3390/nu17213409 (PMC12608203; doi:10.3390/nu17213409)
Supplement: Supplementary file 1 [file nutrients-17-03409-s001.zip › supplementary table s1.pdf]

Supplementary Table S1. PKU severity of patients participating in study.

Patient number	Variants	Variants	PKU severity
**1**	c.1042C>G p.(Leu348Val)	c.1315+1G>A p.?	Classical PKU
**2**	c.1222C>T p.(Arg408Trp)	c.1223G>A p.(Arg408Gln)	Mild PKU
**3**	c. 1066-11 G>A p. ?	c. 1066-11 G>A p. ?	Classical PKU
**4**	c.782G>A p.(Arg261Gln)	c.896T>G p.(Phe299Cys)	Mild PKU
**5**	n/a	n/a	Classical PKU
**6**	c.558_559del p.(Trp187Glyfs*12)	c.558_559del p.(Trp187Glyfs*12)	Classical PKU
**7**	c.558_559del p.(Trp187Glyfs*12)	c.558_559del p.(Trp187Glyfs*12)	Classical PKU
**8**	n/a	n/a	Classical PKU
**9**	c.727C>T p.(Arg243*)	c.782G>A p.(Arg261Gln)	Mild PKU
**10**	c.754C>T p.(Arg252Trp)	c.754C>T p.(Arg252Trp)	Classical PKU
**11**	n/a	n/a	Mild PKU
**12**	n/a	n/a	Classical PKU
**13**	c. 1066-11 G>A p. ?	c. 912+1 G>A p. ?	Classical PKU
**14**	c.47_48del p.(Ser16*)	c.1222C>T p.(Arg408Trp)	Classical PKU
**15**	n/a	n/a	Classical PKU
**16**	c.1222C>T p.(Arg408Trp)	c.1223G>A p.(Arg408Gln)	Mild PKU
**17**	c.194T>C p.(Ile65Thr)	C.1066-11G>Ap.?	Mild PKU
**18**	c.782G>A p.(Arg261Gln)	c. 912+1 G>A p. ?	Classical PKU
**19**	c.745C>T p.(Leu249Phe)	c.896T>G p.(Phe299Cys)	Classical PKU
**20**	n/a	n/a	Mild PKU
**21**	n/a	n/a	Classical PKU
**22**	c. 1241 A>G	c.117 C>G p.Phe39leu	Mild PKU

**23**	c.727C>T p.(Arg243*)	c.844G>A p.(Asp282Asn)	Classical PKU
**24**	c.1315+1G>A p.?	c.896T>G p.(Phe299Cys)	Classical PKU
**25**	n/a	n/a	Classical PKU
**26**	n/a	n/a	Classical PKU
**27**	n/a	n/a	Mild PKU
**28**	n/a	n/a	Classical PKU
**29**	n/a	n/a	n/a
**30**	c.1241A>G p.(Tyr414Cys)	c.1222C>T	Mild PKU
**31**	n/a	n/a	Mild PKU
**32**	n/a	n/a	Mild PKU
**33**	n/a	n/a	Mild PKU

Abbreviations: n/a: not available; PKU: Phenylketonuria.
