# Supplementary material for: Navigating Adolescence with PKU: Adherence, Metabolic Control, and Wellbeing in a UK Clinical Centre
Source: Nutrients. 2025 Oct 29;17(21):3409. doi: 10.3390/nu17213409 (PMC12608203; doi:10.3390/nu17213409)
Supplement: Supplementary file 1 [file nutrients-17-03409-s001.zip › supplementary table s2.pdf]

Supplementary Table 2. Food neophobia questionnaire results of all the participants in the in study.

Patient	I frequently try new and different foods	I don't trust new foods	I like foods from different countries	I think ethnic food looks too weird to eat	At social events, I will try new foods	I am afraid to eat things I have never had before	I am very particular about the foods I will eat	I will eat almost anything	I like to try new foods when eating out	I am uncomfor-table in new & different situations	I prefer to be at home among familiar surround-dings	I avoid speaking to people I don't know	I feel uneasy in unfami-liar surround-dings	I don't like sitting next to someone I don't know
**1**	6	6	6	6	6	1	1	7	7	3	5	1	3	1
**2**	1	6	2	7	1	7	7	2	3	5	4	2	3	2
**3**	2	6	1	7	2	2	3	6	2	3	5	3	6	6
**4**	7	1	3	3	7	1	1	7	7	1	6	7	7	1
**5**	1	7	1	1	1	7	7	1	1	7	7	7	7	7
**6**	3	2	2	5	1	2	6	2	2	2	2	1	1	2
**7**	3	3	2	7	2	5	6	1	2	4	1	3	3	3

**8**	6	5	2	3	2	5	2	5	2	5	2	2	5	5
**9**	6	2	2	3	5	4	7	3	2	4	3	2	2	7
**10**	4	5	4	4	2	6	4	4	1	5	7	4	5	7
**11**	6	5	5	2	6	6	3	1	2	5	3	5	6	6
**12**	3	2	4	4	2	2	2	7	4	5	5	3	2	2
**13**	3	6	2	6	3	6	5	1	5	2	2	1	1	1
**14**	4	3	4	4	3	4	1	5	4	2	1	2	1	1
**15**	2	3	4	6	2	6	6	3	2	3	3	3	3	3
**16**	4	2	7	3	2	3	3	1	3	1	1	1	1	1
**17**	3	5	4	1	4	3	2	3	4	3	1	3	3	7
**18**	6	6	2	4	7	2	2	7	5	1	1	1	1	1
**19**	2	4	1	1	1	6	2	5	1	6	6	6	6	6
**20**	5	5	5	5	7	6	1	7	7	1	3	7	7	7
**21**	5	7	5	7	3	7	3	4	3	5	5	7	3	7

22	2	5	2	2	2	3	2	7	3	2	6	2	6	4
23	5	3	2	7	3	3	1	3	4	6	3	1	2	3
24	3	2	7	7	3	2	3	6	7	2	1	1	3	2
25	6	1	7	7	7	1	1	7	7	3	6	3	3	1

Scale: 1 = always; 2 =mostly; 3=sometimes; 4=unsure; 5 = rarely; 6=almost never; 7=never.
